# Anaphylaxis Presenting as Uvulitis

**DOI:** 10.7759/cureus.17853

**Published:** 2021-09-09

**Authors:** Lilly Nguyen, Thor S Stead, Carlos Lopez Ortiz, Rita Gillespie, Latha Ganti

**Affiliations:** 1 Emergency Medicine, Trinity Preparatory School, Winter Park, USA; 2 Medicine, The Warren Alpert Medical School of Brown University, Providence, USA; 3 Emergency Medicine, Ocala Regional Medical Center, Ocala, USA; 4 Emergency Medicine, Envision Physician Services, Plantation, USA; 5 Emergency Medicine, University of Central Florida College of Medicine /HCA Graduate Medical Education, Ocala, USA; 6 Emergency Medicine, Lakeland Regional Health, Lakeland, USA; 7 Emergency Medicine, University of Central Florida College of Medicine, Orlando, USA; 8 Emergency Medicine, HCA Healthcare Graduate Medical Education Consortium Emergency Medicine Residency Program of Greater Orlando, Orlando, USA

**Keywords:** anaphylaxis, airway emergency, emergency medicine, uvulitis, allergy and anaphylaxis

## Abstract

The authors present a case of a young man who woke up with uvular swelling resulting in a severely narrowed airway. He had ingested peanut butter the prior night but was unaware of any allergies. He was treated with epinephrine, diphenhydramine, and methylprednisolone which resulted in resolution of the airway compromise. The authors discuss the mechanism of anaphylaxis and the emergency management of this life-threatening condition.

## Introduction

Anaphylaxis is a life-threatening immune hypersensitivity reaction frequently triggered by various antigens such as tree nuts and insect bites [1]. Upon exposure to allergens, the misactivated immune system initiates the production of immunoglobulin E (IgE) and immunoglobulin G (IgG) antibodies that bind to mast cells, the body’s first line of immune defense. Mast cells travel to the affected peripheral region to attack interpreted threats, resulting in symptoms including tissue, skin, and mucosal texture changes [2]. While the role of mast cells is immunomodulation, when they localize in inappropriate peripherals, patients can experience life-threatening repercussions; most notably jeopardized airway, impaired circulation, and upset gastrointestinal processes [3]. Monophasic anaphylaxis occurs in one wave of symptoms. Biphasic anaphylaxis can be much more deadly and is followed by a second recurrence of symptoms after the patient appears to have recovered [4].

The possibility that presenting symptoms are an indicator of anaphylaxis can be evaluated using the National Institute of Allergy and Infectious Disease (NAIAD) Criterion for anaphylaxis. If at least one of the following 3 criteria are met, a diagnosis of anaphylaxis can be made: 1) Acute onset of affected skin or mucosal tissue with respiratory compromise or a decrease in blood pressure; 2) Involvement of 2 of the following after an exposure: skin or mucosal manifestations, a decrease in blood pressure, gastrointestinal tract upset, or respiratory compromise; 3) Hypotension with >30% decrease in systolic blood pressure [5].

## Case presentation

A 36-year-old male presented to the emergency department (ED) with the chief complaint of a swollen uvula. The patient went to bed fine the previous night and woke up to an uvula that was so swollen it was hard to breathe. This happened to him once before and it resolved with prednisone and ampicillin. The patient stated he was in his usual state of good health. He denied any fevers, chills, chest pain, nausea, vomiting, diarrhea, abdominal pain, headache, or urinary symptoms. He only complained of the fullness in his throat. He did not endorse pain or feelings of fear or panic. As far as he knew, he was not allergic to anything. He stated that the last thing he had before he went to bed was peanut butter. However, he had previously eaten peanut butter many times without any problems. The patient’s daily medication included citalopram, dextroamphetamine, and levothyroxine daily for depression, attention deficit disorder, and hypothyroidism, respectively. None of these medications were new for him.

**Figure 1:**
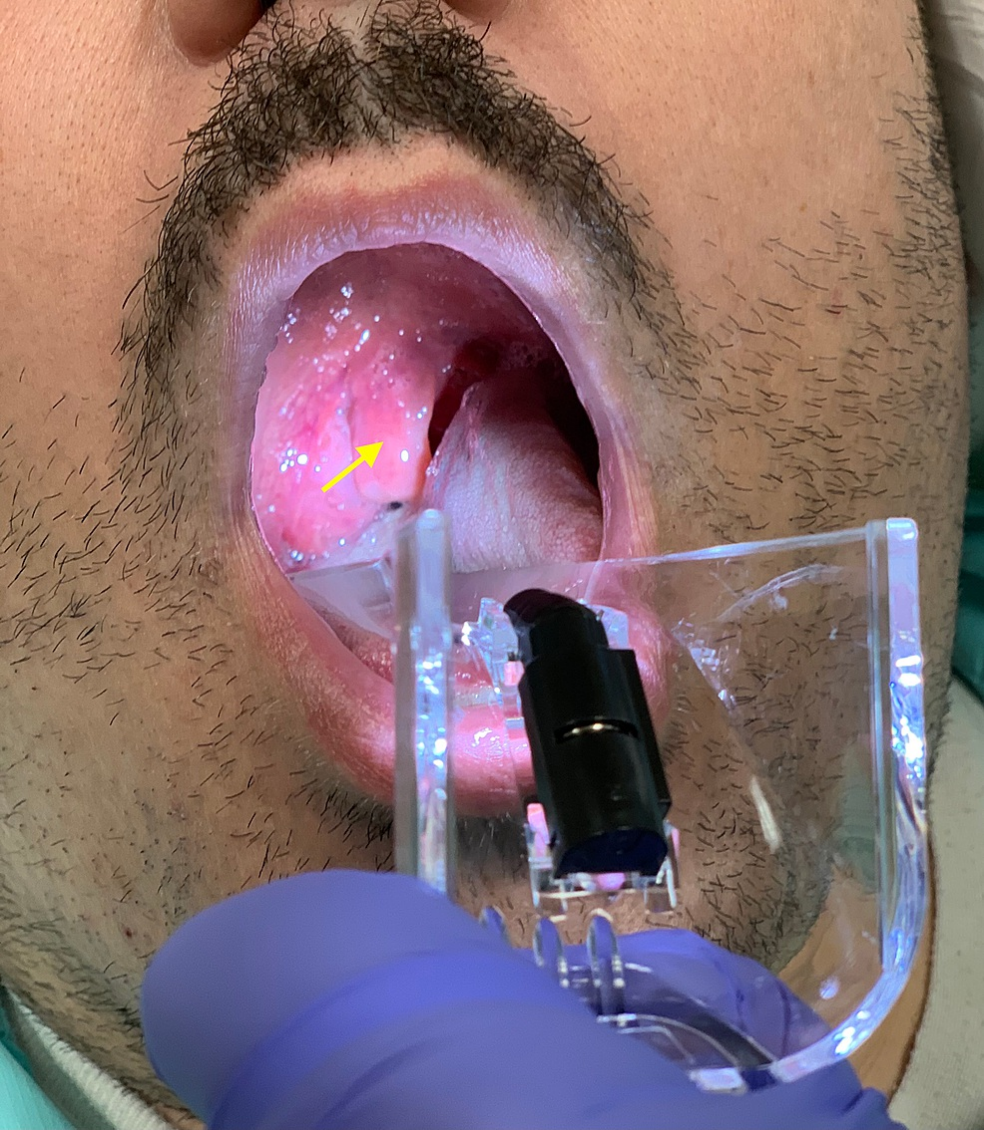
Clinical photograph demonstrating the patient’s grossly edematous uvula (arrow)

His vital signs in the ED were blood pressure 132/86 mmHg, temperature 97.8^0^F, pulse 77 beats/min, respiration rate 16 breaths/minute, and saturating at 96% on room air. Physical examination revealed equal bilateral breath sounds and regular heart rate and rhythm. He was alert and oriented. Oropharynx revealed a grossly edematous uvula (Figure 1).

**Figure 2:**
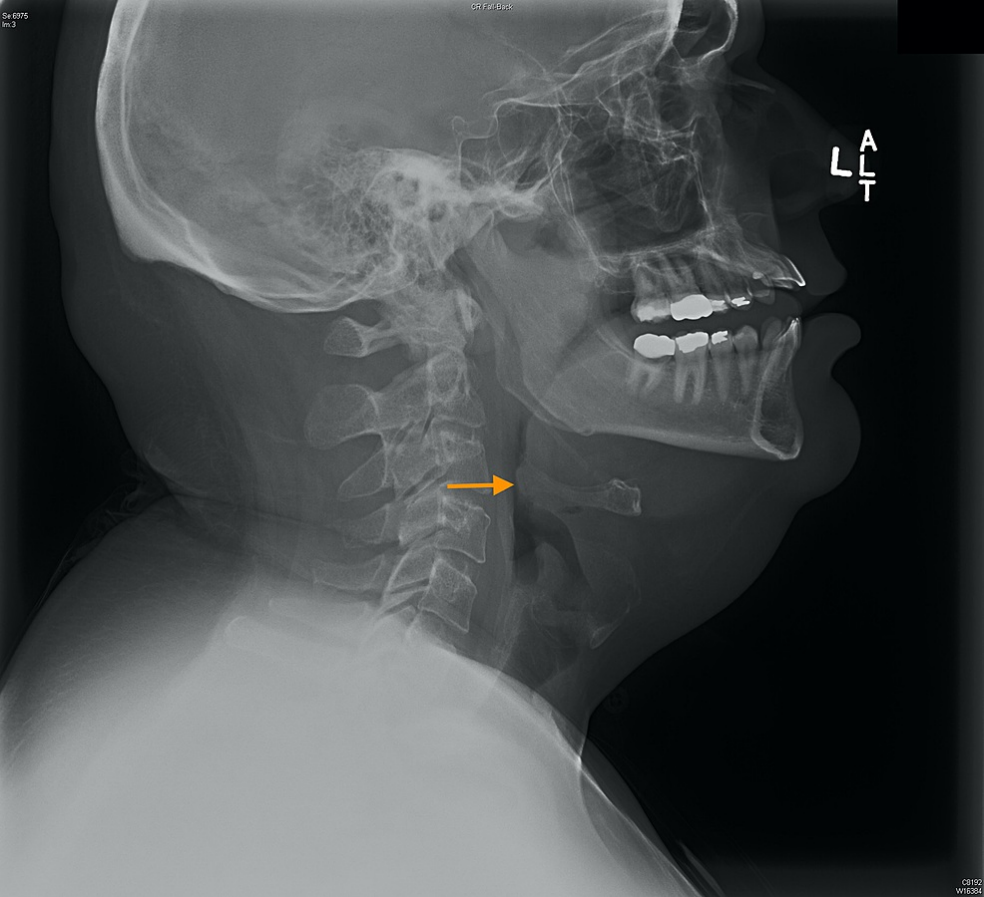
Lateral view radiograph of soft tissue of neck demonstrating severely narrowed airway (arrow)

Examination of the neck was atraumatic and supple, with full range of motion. There was no meningismus, adenopathy, or swelling. There was no crepitus, mass, carotid bruit, or tracheal deviation. No abnormalities were noted in the abdomen or extremities. Plain radiograph of the soft tissue of the neck showed soft tissue enlargement of the nasopharynx due to uvular swelling with severe narrowing of the airway, without any prevertebral soft tissue swelling (Figure 2).

**Figure 3:**
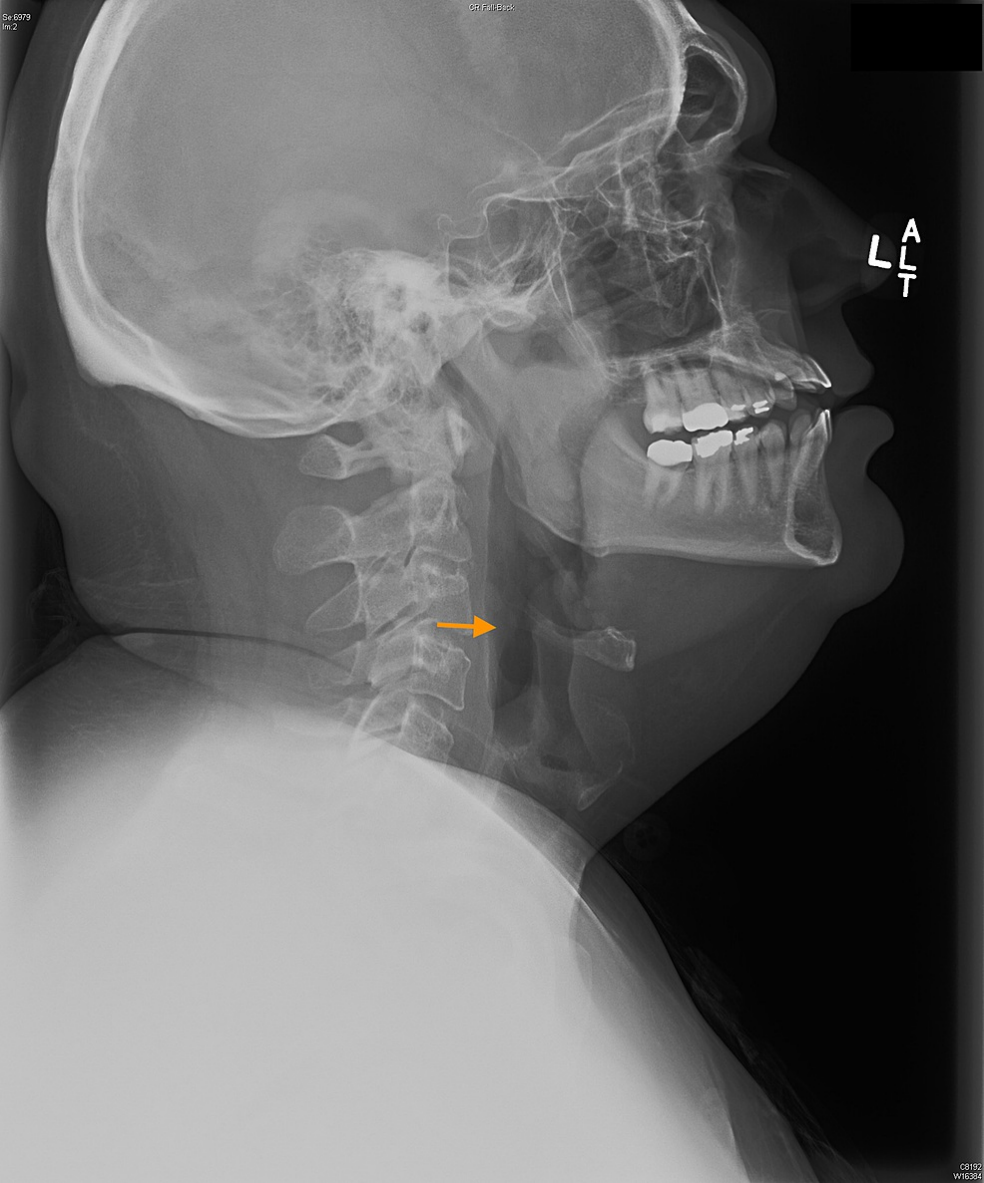
Lateral view radiograph of soft tissue of neck 2 hours and 37 minutes later demonstrating much-improved airway (arrow)

The patient was treated promptly for anaphylaxis with 0.3 mg 1:1000 intramuscular epinephrine, 25 mg intravenous diphenhydramine, and 125 mg intravenous methylprednisolone. He also received 3g of intravenous ampicillin-sulbactam in case the uvulitis was bacterial in nature. Repeat radiograph of his neck two and a half hours later revealed a decrease of the previously seen nasopharyngeal soft tissue swelling and significant decrease in the airway narrowing. The epiglottis and aryepiglottic folds appeared normal. There was no enlargement of the adenoids or palatine tonsils. There was again no prevertebral soft tissue swelling (Figure 3).

Following resolution of the airway compromise, the patient was discharged home with prescriptions for an epinephrine auto-injector, prednisone 50 mg once daily for 5 days, and amoxicillin-clavulanate 875-125 mg twice a day for 7 days. 

The patient’s prompt discharge following resolution of the airway narrowing on x-ray after a single dose of epinephrine is consistent with the 2021 United Kingdom Resuscitation Council guidelines for anaphylaxis [6]. The patient was followed up by telephone 4 months later and continued to be in good health, with no further allergic reactions or anaphylactic episodes.

## Discussion

Adult-onset food allergy has become increasingly prevalent in recent years; 40-60% of allergy cases initiate in adulthood [7,8]. In this case, hyperreactivity to peanut butter ingestion presented through edema of the uvula, one of the less common symptoms of anaphylaxis. However, uvulitis can be dangerous as it can lead to impending airway obstruction [9]. 

**Figure 4:**
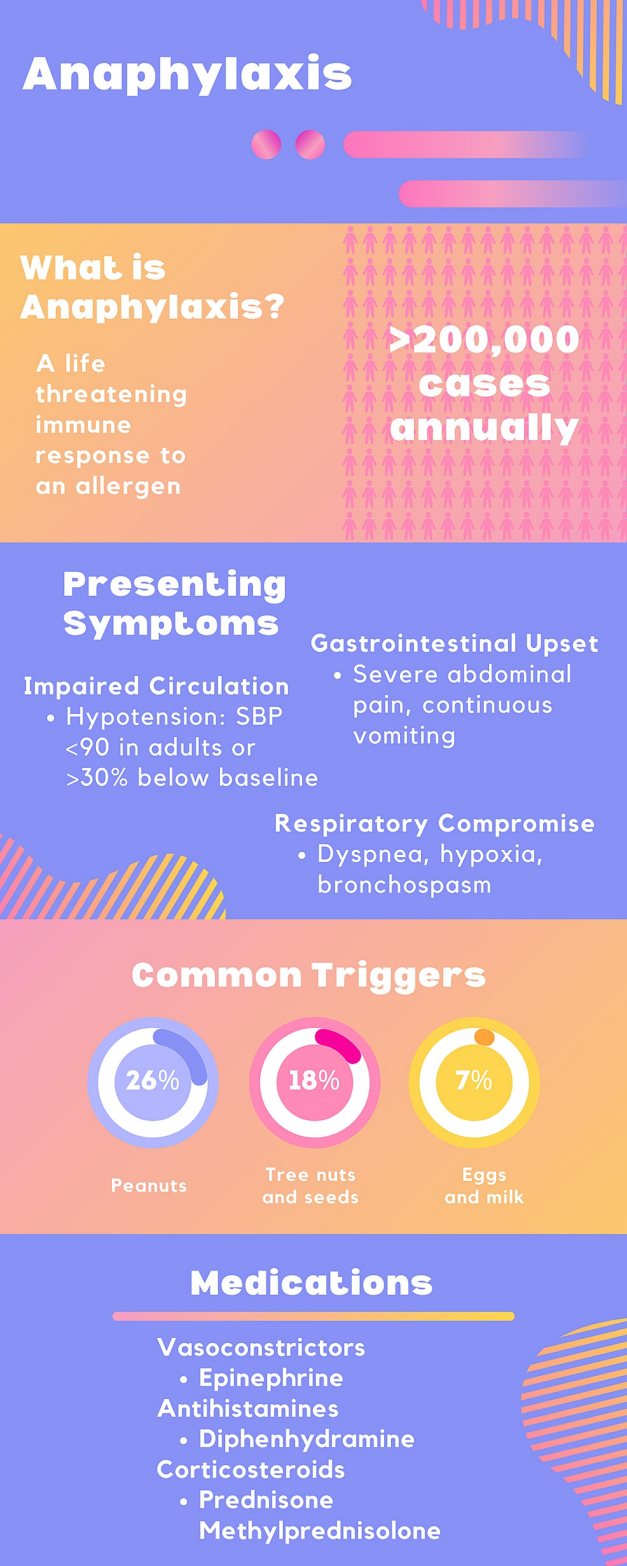
Infographic summarizing anaphylaxis

Emergency management of anaphylaxis consists of epinephrine, antihistamines, corticosteroids, and fluid resuscitation (Figure 4). 

Epinephrine is the first line of treatment for anaphylaxis. Failing to administer timely epinephrine can result in progression to biphasic anaphylaxis or death [6]. During an allergic reaction, vasodilating histamines increase vascular permeability, resulting in inflammation. Second-line treatments for anaphylaxis include antihistamines and glucocorticoids. Antihistamines are administered in an attempt to down‐regulate the allergic response and minimize the clinical impact of histamine release, although a Cochrane review was unable to find strong evidence to support antihistamine administration for anaphylaxis. [10]. H1-antihistamines block receptors on mast cells, smooth muscle, and nerves. H2-antihistamines block receptors in the gastrointestinal tract and are given to decrease gastric acid production and prevent cross reactivity. In ED patients experiencing anaphylaxis, administration of antihistamines correlates with lower chances of progression to biphasic anaphylaxis [11]. Glucocorticoids dampen inflammatory processes and maturation and activation of mast cells [12].

## Conclusions

This case describes a patient who presented with uvulitis significant enough to cause marked airway compromise. Emergency management of this condition as anaphylaxis resulted in prompt resolution of his symptoms without the development of a biphasic component. Anaphylaxis remains an under-recognized problem, despite being life-threatening. Emergency management of this manifestation consists of epinephrine as first-line pharmacologic therapy, followed by antihistamines and glucocorticoids as second-line therapy despite solid evidence to back their efficacy.
